# Computed Tomography‐Based Habitat Analysis for Prognostic Stratification in Colorectal Liver Metastases

**DOI:** 10.1002/cai2.70000

**Published:** 2025-03-12

**Authors:** Chaoqun Zhou, Hao Xin, Lihua Qian, Yong Zhang, Jing Wang, Junpeng Luo

**Affiliations:** ^1^ Department of Pathology, Huaihe Hospital Henan University Kaifeng Henan China; ^2^ Department of Radiology, Huaihe Hospital Henan University Kaifeng Henan China; ^3^ Department of Biological Therapy Henan Provincial Cancer Hospital Zhengzhou Henan China; ^4^ Department of General Surgery First Medical Center of PLA General Hospital Beijing China; ^5^ Translational Medical Center, Huaihe Hospital Henan University Kaifeng Henan China

**Keywords:** colorectal liver metastases, CT imaging, habitat analysis, prognostic stratification, radiomics, The Cancer Imaging Archive

## Abstract

**Background:**

Colorectal liver metastasis (CRLM) has a poor prognosis, and traditional prognostic models have certain limitations in clinical application. This study aims to evaluate the prognostic value of CT‐based habitat analysis in CRLM patients and compare it with existing traditional prognostic models to provide more evidence for individualized treatment of CRLM patients.

**Methods:**

This retrospective study included 197 patients with CRLM whose preoperative contrast‐enhanced CT images and corresponding DICOM Segmentation Objects (DSOs) were obtained from The Cancer Imaging Archive (TCIA). Tumor regions were segmented, and habitat features representing distinct subregions were extracted. An unsupervised K‐means clustering algorithm classified the tumors into two clusters based on their habitat characteristics. Kaplan–Meier analysis was used to evaluate overall survival (OS), disease‐free survival (DFS), and liver‐specific DFS. The habitat model's predictive performance was compared with the Clinical Risk Score (CRS) and Tumor Burden Score (TBS) using the concordance index (C‐index), Integrated Brier Score (IBS), and time‐dependent area under the curve (AUC).

**Results:**

The habitat model identified two distinct patient clusters with significant differences in OS, DFS, and liver‐specific DFS (*p* < 0.01). Compared with CRS and TBS, the habitat model demonstrated superior predictive accuracy, particularly for DFS and liver‐specific DFS, with higher time‐dependent AUC values and improved model calibration (lower IBS).

**Conclusions:**

CT‐based habitat analysis captures spatial tumor heterogeneity and provides enhanced prognostic stratification in CRLM. The method outperforms conventional models and offers potential for more personalized treatment planning.

## Graphical abstract

Workflow for CT‐based habitat analysis and prognostic stratification in colorectal liver metastases. (a) Preoperative contrast‐enhanced CT images were acquired in the portal venous phase, and liver lesions were manually segmented to delineate the tumor regions. (b) Segmented tumor regions were analyzed using the volume of interest (VOI) for habitat imaging, extracting features representing distinct subregions within the tumor based on intensity, texture, and enhancement patterns. (c) An unsupervised K‐means clustering algorithm was applied to classify the tumors into two distinct clusters based on their habitat characteristics. The optimal number of clusters was determined using the Elbow method and silhouette analysis. (d) Kaplan–Meier survival curves for OS, DFS, and liver‐specific DFS were generated based on the clustering results, demonstrating significant differences in patient prognosis and enabling prognostic stratification.

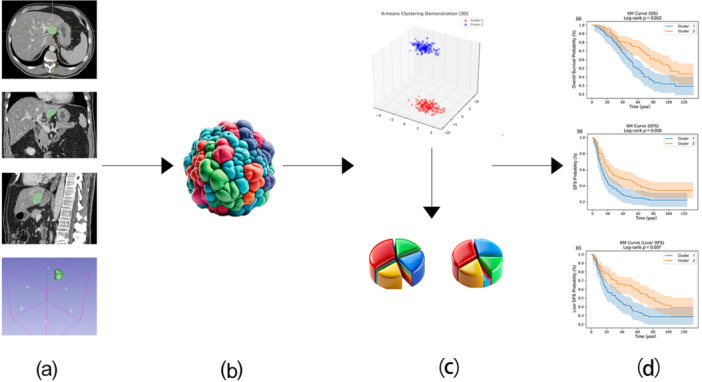

**Abbreviations**
AUC: area under the curve
CRLM: colorectal liver metastases
CRS: clinical risk score
DFS: disease‐free survival
DSO: DICOM segmentation object
IBS: integrated brier score
KVP: kilovolt peak
MDCT: multidetector computed tomography
NRI: net reclassification improvement
OS: overall survival
PVE: portal vein embolization
ROC: receiver operating characteristic
TBS: tumor burden score
TCIA: The Cancer Imaging Archive
VOI: volume of interest

## 1 Background

Colorectal cancer (CRC) is the third most prevalent cancer globally and the third leading cause of cancer‐related mortality, contributing significantly to the worldwide healthcare burden [1]. Colorectal liver metastases (CRLM) are present in nearly 30% of patients diagnosed with colorectal cancer, and their management poses significant clinical challenges [2]. Despite advances in surgical and systemic therapies, the prognosis for patients with CRLM varies greatly, and improved risk stratification is urgently required to guide treatment decisions and optimize outcomes [3].

Traditional prognostic models, such as the clinical risk score (CRS) and tumor burden score (TBS), have been widely used to assess patient risk and predict survival outcomes in CRLM [4, 5, 6]. However, although these models rely on clinical and pathological factors, they often fail to capture the complex biological behavior of metastatic tumors, which can differ significantly among patients, even between those with similar clinical presentations. Tumor heterogeneity, particularly at the level of the microenvironment, is increasingly being recognized as a critical factor reflecting cancer progression, metastasis, and treatment resistance [7, 8]. However, current prognostic tools lack the ability to adequately account for this heterogeneity.

Radiomics, which involves the extraction of quantitative features from medical images, has emerged as a promising tool for the noninvasive assessment of tumor biology [9]. By analyzing variations in texture, intensity, and other image‐derived features, radiomics can provide insights into the underlying tumor microenvironment and its spatial heterogeneity [10, 11]. Recent studies have demonstrated the potential of radiomics to improve prognostic models through the incorporation of imaging biomarkers that reflect the biology of CLRM [12, 13, 14]. However, while traditional radiomics has proven valuable for extracting imaging features that correlate with tumor biology, the techniques have primarily focused on the analysis of global whole‐tumor characteristics [15], whereas tumors are inherently heterogeneous with distinct subregions that may have different biological behaviors such as varying levels of hypoxia, angiogenesis, or metabolic activity [16]. This intratumor heterogeneity, which global radiomics techniques may not fully capture, often drives treatment resistance and disease progression [17].

Habitat imaging addresses this limitation by segmenting tumor into biologically distinct subregions, or “habitats,” on the basis of imaging data [18]. These habitats can reveal crucial insights into the spatial variations within a tumor, allowing for a more nuanced understanding of its complexity. This detailed subregional analysis is crucial for identifying areas that may respond differently to therapies or that represent more aggressive disease components, thus offering a more personalized and accurate prognosis [19].

This study aimed to evaluate the utility of CT‐based habitat analysis in the prognostic stratification of patients with CRLM. By extracting and analyzing habitat features from preoperative contrast‐enhanced CT images, we sought to develop a CT‐based habitat model that could more accurately predict overall survival (OS), disease‐free survival (DFS), and liver‐specific DFS than more traditional models such as CRS and TBS. We hypothesized that the incorporation of tumor habitat features would provide a more comprehensive understanding of tumor heterogeneity, thereby improving risk stratification and enabling more personalized treatment planning for patients with CRLM.

## 2 Methods

### 2.1 Study Design and Patient Population

This retrospective study included 197 patients diagnosed with colorectal liver metastases (CRLM) for whom preoperative CT images and corresponding DICOM Segmentation Objects (DSOs) were obtained from The Cancer Imaging Archive (TCIA), a publicly accessible database. This data set contains both raw DICOM CT images and DICOM‐SEG files for quantitative habitat analysis, ensuring consistency across all patients [20]. The images were acquired at a single institution, and the data set includes demographic, pathological, and survival data.

The inclusion criteria were as follows: (1) pathologically confirmed resected CRLM; (2) available data from pathologic analysis of both the hepatic tumor and the underlying nontumoral liver parenchyma; and (3) preoperative conventional multidetector computed tomography (MDCT) scans performed within 6 weeks of hepatic resection.

Patients with mortality within 90 days of imaging, less than 24 months of follow‐up, or those who underwent hepatic artery infusion (HAI) for chemotherapy were excluded. Additionally, patients who underwent local tumor ablation, more than three wedge resections, or had no visible tumor on preoperative imaging were excluded to ensure accurate tumor burden assessment.

The CT images were originally extracted from the institution's Picture Archiving and Communication System (PACS) and underwent deidentification in compliance with Health Insurance Portability and Accountability Act regulations. These deidentified images, along with corresponding DSOs, were subsequently uploaded to TCIA. The tumors, liver, and vessels were segmented using Scout Liver software (Pathfinder Technologies Inc., TN, USA), with postoperative imaging and/or pathology reports used to delineate surgical resection lines. The resulting segmentation masks were converted into DICOM‐SEG format using the DCMQI open‐source software, ensuring compatibility and ease of use for subsequent quantitative analysis. The study used pre‐annotated tumor masks from a public data set, which were originally generated by the institution through automatic segmentation and subsequently uploaded to TCIA. These masks covered the entire tumor area, whether or not the patient had a single lesion or multiple lesions. To ensure accuracy and precision, manual corrections were made, especially in cases where tumors were adjacent or overlapping.

### 2.2 Imaging Acquisition and Preprocessing

**Figure 1:**
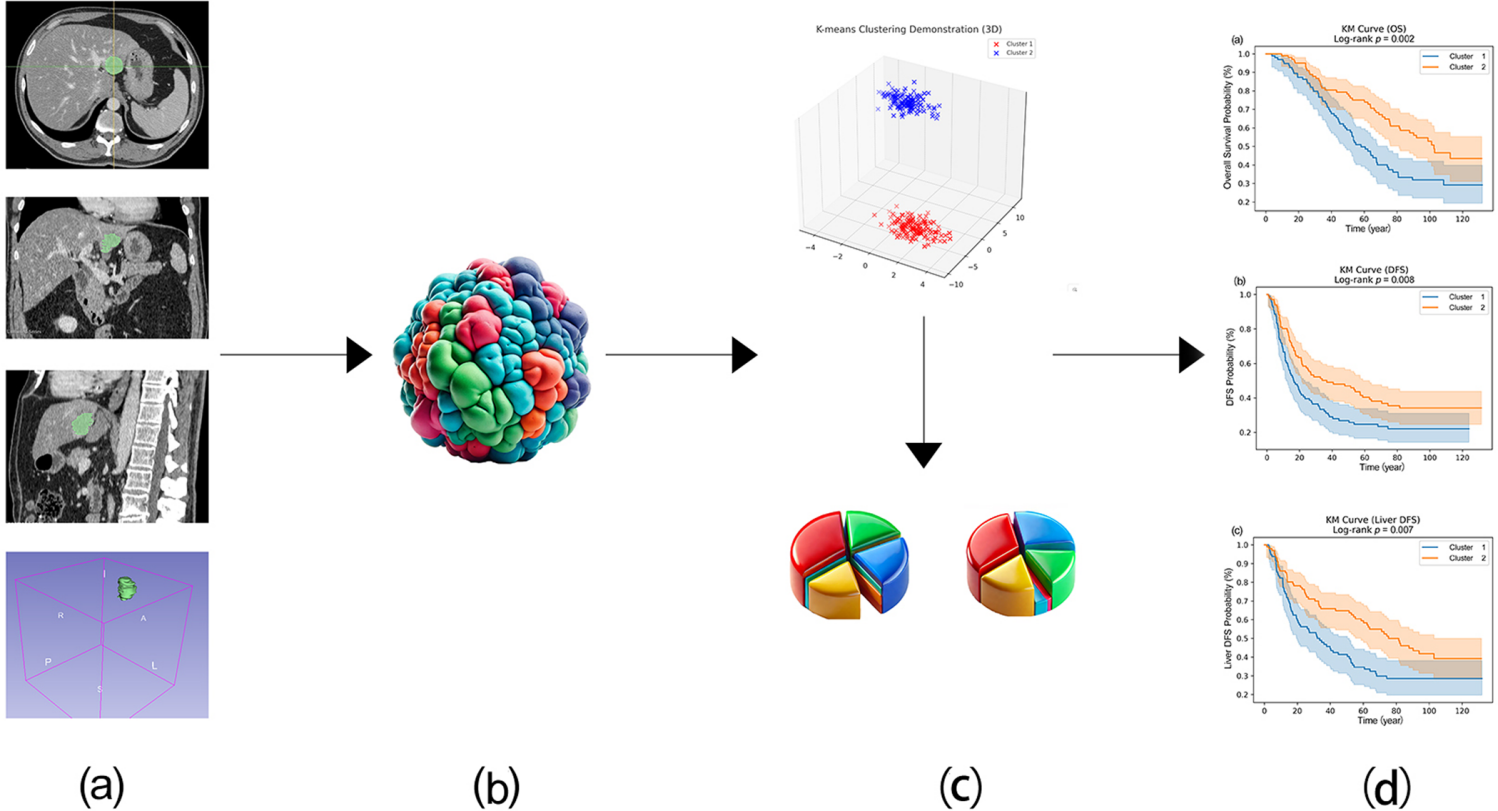
Workflow for CT‐based habitat analysis and prognostic stratification in colorectal liver metastases. (a) Preoperative contrast‐enhanced CT images were acquired in the portal venous phase, and liver lesions were manually segmented to delineate the tumor regions. (b) Segmented tumor regions were analyzed by defining a VOI that encompassed the entire tumor. Quantitative features, including intensity, texture, and enhancement patterns, were extracted voxel‐wise from the VOI to capture spatial heterogeneity. These features were used for clustering analysis to identify distinct tumor habitats. (c) An unsupervised K‐means clustering algorithm was applied to classify the tumors into two distinct clusters based on their habitat characteristics. The optimal number of clusters was determined using the elbow method and silhouette analysis. (d) Kaplan–Meier survival curves for OS, DFS, and liver‐specific DFS, along with time‐dependent ROC curves, were generated based on the clustering results. These analyses revealed significant differences in patient prognosis and supported effective prognostic stratification. DFS, disease‐free survival; OS, overall survival; ROC, receiver operating characteristic; VOI, volume of interest.

Preoperative contrast‐enhanced CT scans were acquired in the portal venous phase to maximize the visibility of liver lesions (Figure 1a). The CT scans were acquired using standardized protocols, with slice thicknesses ranging from 2.5 to 7.5 mm, tube voltages (KVP) between 120.0 and 140.0 kVp, and tube currents ranging from 83.0 to 760.0 mA. The pixel spacing, which determines the spatial resolution, varied between 0.609 and 0.977 mm. To account for variations in imaging acquisition parameters, we standardized all CT scan images before analysis to ensure consistency and minimize their potential impact on the results.

### 2.3 Definition of Survival Indicators

OS was defined as the time from the initiation of treatment or diagnosis to the time of death. OS is an important indicator for assessing overall patient prognosis, reflecting the overall effectiveness of treatment and the patient's long‐term survival. DFS was defined as the time from the end of treatment or surgery to the occurrence of disease recurrence or any other related event. DFS reflects the patient's posttreatment quality of life and disease control and is primarily used to assess the risk of disease recurrence after treatment. Liver‐specific DFS was defined as the time from the end of treatment until the recurrence or metastasis of liver lesions, excluding recurrence or metastasis at other sites. Liver‐specific DFS is particularly useful for assessing the prognosis in patients with liver metastasis because it provides a more precise prediction of disease control in the liver. Although both DFS and liver‐specific DFS are used to measure disease‐free survival, their scope of application and clinical significance differ. DFS is a broad indicator that includes all types of disease recurrence, including recurrence or metastasis at any site in the body, and reflects the overall disease control status of the patient after treatment. By contrast, liver‐specific DFS is a specific survival indicator for patients with liver metastasis, focusing on the recurrence of lesions in the liver. This makes liver‐specific DFS better targeted for evaluating the prognosis of patients with liver metastasis, providing more valuable insights for treatment decisions. Combining these two indicators offers a more comprehensive prognostic assessment for clinical decision‐making, aiding in the development of personalized treatment plans.

### 2.4 Tumor Segmentation and Habitat Feature Extraction

Tumor segmentation masks were provided in the DICOM‐SEG format for all patients. Using these pre‐segmented tumor regions, habitat features were extracted from the volume of interest (VOI). The analysis focused on identifying distinct subregions within each tumor based on intensity, texture, and enhancement patterns, with these representing the heterogeneous nature of the tumor microenvironment (Figure 1b). Habitat features capture spatial variations across the tumor and are crucial for understanding the biological diversity within tumor regions.

### 2.5 Tumor Burden Score (TBS) Definition

The TBS was calculated for each patient to provide a measure of tumor load. TBS combines both the size of the largest tumor and the number of liver metastases and is defined by Equation (1):

(1) $$\mathrm{TBS}=\sqrt{d_{\max}^{2}+n^{2}},$$

where *d*_max_ represents the diameter of the largest liver metastasis (in cm), and *n* is the total number of liver metastases. TBS provides a continuous scale for assessing tumor burden, with higher scores indicating a larger tumor burden. This score was shown to have prognostic significance in colorectal liver metastases, allowing for a refined evaluation of tumor aggressiveness.

### 2.6 Habitat Analysis and Clustering

Unsupervised machine learning techniques were used to analyze the habitat features and identify distinct tumor clusters. A K‐means clustering algorithm was applied to classify tumors into two distinct clusters on the basis of their habitat characteristics (Figure 1c). Before clustering, the habitat features were normalized using *Z*‐score normalization, and dimensionality reduction was performed to facilitate clustering. The optimal number of clusters was determined using the elbow method and silhouette analysis, in which a silhouette score of 0.96 supported the selection of two clusters (Supporting Information: Figure S1).

### 2.7 Statistical Analysis

Continuous variables are summarized as mean with standard deviation or median with interquartile range, depending on their distribution. Student's *t*‐test was used to compare normally distributed continuous variables, while the Mann–Whitney *U* test was applied for non‐normally distributed variables. Categorical variables are presented as frequencies and percentages, and comparisons were made using the *χ* ^2^ test or Fisher's exact test where appropriate.

For survival analysis, including OS, DFS, and liver‐specific DFS, Kaplan–Meier curves were generated to estimate survival probabilities, and the log‐rank test was used to compare survival differences between clusters. Additionally, multivariate Cox proportional hazards regression analysis was conducted to calculate hazard ratios (HRs) with 95% confidence intervals (CIs) for relevant prognostic factors, including habitat features, fibrosis, tumor size, and other clinical variables. This analysis allowed for the assessment of the independent prognostic significance of each factor.

Several metrics were used to compare the predictive performance of the habitat‐based model with conventional prognostic models such as the CRS and TBS. The concordance index (C‐index) was calculated to assess each model's discriminative ability, while the Integrated Brier Score (IBS) was computed to evaluate model calibration and the accuracy of survival predictions over time. Furthermore, net reclassification improvement (NRI) and integrated discrimination improvement (IDI) were applied to quantify the models' ability to improve patient classification and enhance discriminatory power, respectively.

Time‐dependent receiver operating characteristic (ROC) analysis was performed to assess the temporal predictive accuracy of the models over a 10‐year period. The area under the curve (AUC) was calculated at multiple time points to evaluate the performance of the models in the prediction of OS, DFS, and liver‐specific DFS.

All statistical analyses were conducted using R software (version 4.4.1) and Python (version 3.11.7). *p* < 0.05 was considered statistically significant for all tests.

## 3 Results

### 3.1 Patient Characteristics and Cluster Analysis

**Figure 2:**
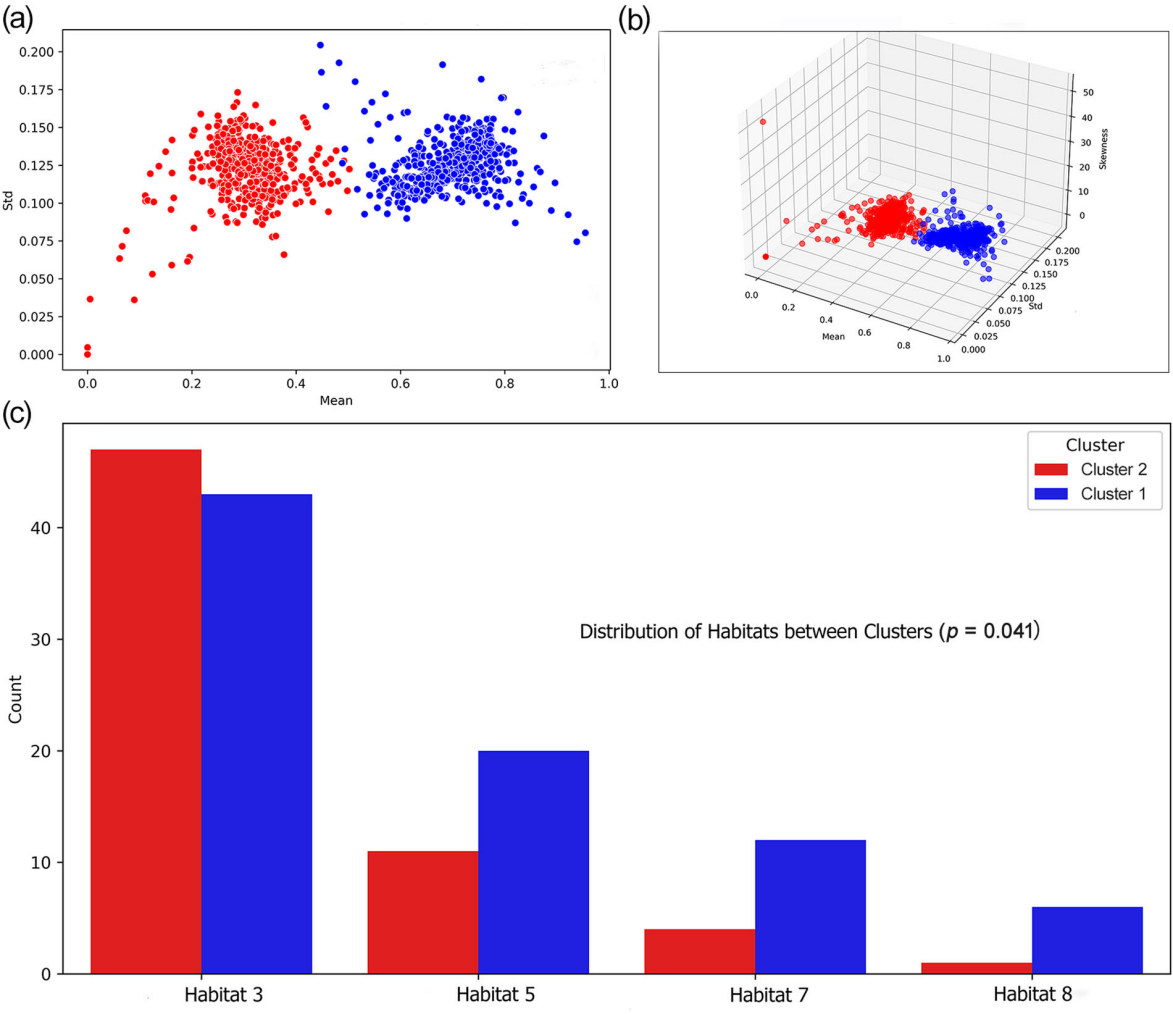
Cluster analysis and habitat distribution in colorectal liver metastases. (a) Scatter plot displaying the distribution of tumors in two distinct clusters (Cluster 2 in red and Cluster 1 in blue) based on mean and standard deviation (Std) of habitat features extracted from the VOI analysis. (b) Three‐dimensional scatter plot showing clustering results using three habitat features (mean, Std, and skewness), illustrating a clear separation between Cluster 2 (red) and Cluster 1 (blue). (c) Bar chart showing the distribution of specific habitats (Habitat 3, Habitat 5, Habitat 7, and Habitat 8) in the two clusters. Significant differences were observed between the clusters (*p* = 0.041), highlighting the distinct habitat characteristics within each cluster.

The study included 197 patients with colorectal liver metastases (CRLM) who were categorized into two distinct clusters based on habitat analysis of radiomics features extracted from preoperative CT. Cluster 1 consisted of 96 patients, while Cluster 2 included 101 patients. Baseline characteristics, including age, gender distribution, BMI, comorbidities, and tumor features, were comparable between the two clusters (all *p* > 0.05). This suggests that the two groups were similar in clinical presentation, allowing for a meaningful comparison of survival outcomes (Figure 2). Detailed baseline characteristics are presented in Table 1, which confirms no significant differences between the clusters across most clinical and pathological factors, including fibrosis, chemotherapy treatment, and metastatic disease burden.

**Table 1:** Baseline characteristics and clinical outcomes of patients in two clusters.

Variable	Cluster 1 *n* = 96^a^	Cluster 2 *n* = 101^a^	*p* b
Age (years)	59 ± 13	60 ± 12	> 0.900
Sex			0.785
Male	56/96 (58)	61/101 (60)	
Female	40/96 (42)	40/101 (40)	
Major comorbidity	57/96 (59)	52/101 (51)	0.331
BMI (kg/m^2^)	27.2 ± 4.8	27.5 ± 5.1	> 0.900
Node‐positive primary	33/96 (34)	36/101 (36)	0.885
Synchronous CRLM	56/96 (58)	55/101 (54)	0.613
Multiple metastases	57/96 (59)	57/101 (56)	0.706
Clinical risk score			0.058
Low	48/77 (62)	69/91 (76)	
High	29/77 (38)	22/91 (24)	
Maximum tumor size (cm)	3.62 ± 2.69	3.36 ± 2.44	0.712
Bilobar disease	40/96 (42)	46/101 (46)	0.604
Extrahepatic disease	10/96 (10)	7/101 (7)	0.367
Chemotherapy before liver resection	65/96 (68)	57/101 (56)	0.109
Preoperative PVE	12/96 (13)	11/101 (11)	0.732
Steatosis (yes/no)	33/96 (34)	35/101 (35)	> 0.900
Presence of sinusoidal dilatation	14/96 (15)	12/101 (12)	0.608
Total response (percent)	0.56 ± 0.23	0.53 ± 0.23	0.484
Necrosis (percent)	0.33 ± 0.25	0.28 ± 0.22	0.217
Fibrosis (percent)	0.19 ± 0.21	0.18 ± 0.20	0.803
Fibrosis > 40%	13/95 (14)	11/99 (11)	0.596
Overall survival (months)	59 ± 34	74 ± 35	0.004
Months to DFS progression	35 ± 37	47 ± 40	0.006
Progression or recurrence (liver only)	47/96 (49)	34/101 (34)	0.029
Months to liver DFS progression	46 ± 39	61 ± 39	0.009
Tumor burden score			0.587
Low	29/96 (30)	25/101 (25)	
Medium	62/96 (65)	69/101 (68)	
High	5/96 (5)	7/101 (7)	

*Note:* Some data in the table are missing due to the lack of recording or incompleteness of the relevant information in the original database.

Abbreviations: CRLM, colorectal liver metastase; DFS, disease‐free survival; OS, overall survival; PVE, portal vein embolization.

^a^ Mean ± SD; *n* (%).

^b^ Wilcoxon rank sum test; Pearson's *χ* ^2^ test.

### 3.2 Survival Outcomes

Significant differences in survival outcomes were observed between the two clusters.

**Figure 3:**
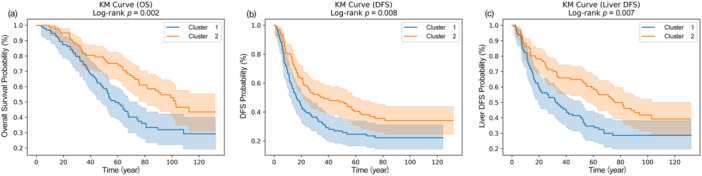
Kaplan–Meier survival curves comparing prognosis between two tumor clusters in colorectal liver metastases. (a) Kaplan–Meier curve for OS, showing significant differences between Cluster 1 (blue) and Cluster 2 (orange) with a log‐rank *p* value of 0.002. (b) Kaplan–Meier curve for DFS, demonstrating a significant difference between the two clusters with a log‐rank *p* of 0.008. (c) Kaplan–Meier curve for liver‐specific DFS, revealing a significant difference between Cluster 1 and Cluster 2 with a log‐rank *p* of 0.007. DFS, disease‐free survival; OS, overall survival.

OS: Patients in Cluster 2 demonstrated significantly longer OS than those in Cluster 1 (*p *= 0.002, Figure 3a). This finding indicates that the radiomics‐based habitat classification effectively differentiated between patient groups with varying survival probabilities.

DFS and liver‐specific DFS: Similar trends were observed for DFS and liver‐specific DFS, with Cluster 2 showing superior outcomes with both metrics (DFS: *p *= 0.008, Figure 3b; liver‐specific DFS: *p *= 0.007, Figure 3c). These findings suggest that the tumor habitat characteristics captured by radiomics are predictive of both overall and liver‐specific disease progression.

### 3.3 Habitat Characterization and Cluster Distribution

The Health Insurance Portability and Accountability Act (HIPAA) ensures the protection of patient data in medical research. In this study, specific tumor habitats—clustered subregions identified from contrast‐enhanced CT images based on variations in intensity, texture, and enhancement patterns—were analyzed. The distribution of these habitats was compared between two distinct clusters, revealing notable differences in habitat composition. Cluster 2 exhibited a higher prevalence of certain habitat types (e.g., Habitat 3), while Cluster 1 contained a greater proportion of more aggressive habitats, such as Habitat 5, Habitat 7, and Habitat 8, as illustrated in Figure 2c. These differences in habitat distribution likely contributed to the observed disparities in survival outcomes.

### 3.4 Multivariate Analysis of Prognostic Factors

**Figure 4:**
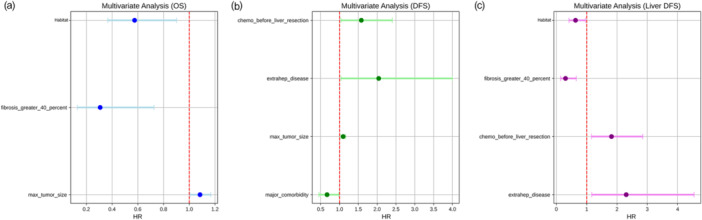
Multivariate analysis of prognostic factors in colorectal liver metastases. (a) Multivariate analysis for OS, illustrating the HRs for significant prognostic factors, including habitat features, fibrosis greater than 40%, and maximum tumor size. (b) Multivariate analysis for DFS, showing HRs for key factors such as chemotherapy before liver resection, extrahepatic disease, maximum tumor size, and major comorbidities. (c) Multivariate analysis for liver‐specific DFS, indicating HRs for prognostic factors including habitat features, fibrosis greater than 40%, chemotherapy before liver resection, and extrahepatic disease. DFS, disease‐free survival; HR, hazard ratio; OS, overall survival.

Multivariate analysis was performed to evaluate the impact of key clinical and radiomics‐derived factors on survival outcomes. This analysis revealed that factors such as habitat characteristics, tumor size, and fibrosis were significant predictors of OS, DFS, and liver‐specific DFS. Figure 4 presents the HRs for each factor for OS, DFS, and liver‐specific DFS, highlighting the strong prognostic significance of habitat features in the survival analysis.

### 3.5 Comparative Analysis of Prognostic Models

**Figure 5:**
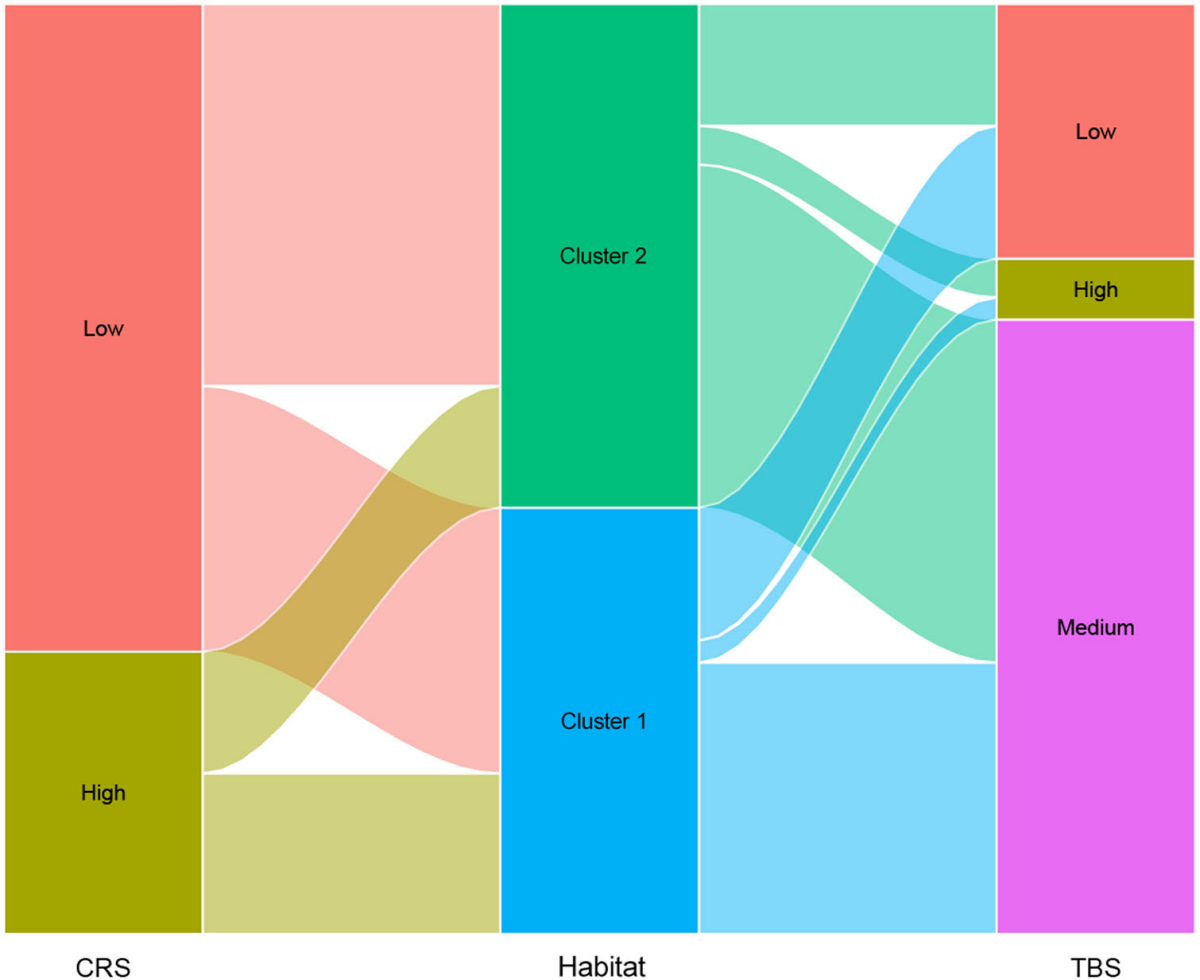
Sankey diagram showing the relationship between CRS, habitat clusters, and TBS in colorectal liver metastases. This Sankey diagram illustrates the flow of patients stratified by low and high CRS and by two habitat clusters (Cluster 1 and Cluster 2), and then further stratified by low, medium, and high TBS. The diagram highlights how habitat clustering provides a distinct stratification between CRS and TBS, revealing potential patterns in tumor biology and clinical outcomes. CRS, clinical risk score; TBS, tumor burden score.

A Sankey diagram (Figure 5) was used to visualize the distribution of patients across the three prognostic models: CRS, TBS, and the Habitat model. This diagram revealed that the Habitat model provided additional stratification detail not fully captured by CRS or TBS. This highlights the additional prognostic value of the habitat model in refining patient risk stratification. The habitat model offers a more nuanced approach by identifying patients whose risk may have been either underestimated or overestimated by traditional models. For instance, patients classified as high risk by the CRS model, but who are classified into Cluster 2 by the Habitat model, may actually have a better prognosis than initially predicted by CRS, and vice versa. This underscores the importance of considering stratified variations in risk, which can provide important information for treatment decisions and adjustments. By incorporating information on the spatial heterogeneity of tumors, the habitat model enhances the predictive accuracy of CRS and TBS, providing a more precise risk assessment. This in turn helps guide personalized treatment strategies. Patients whose risk status changes based on the habitat model may benefit from more tailored management, ultimately improving their prognosis.

### 3.6 Model Performance

The predictive performance of three different prognostic models—CRS, TBS, and the Habitat model—was evaluated using the C‐index and IBS. The Habitat model derived from radiomics features demonstrated a lower IBS (IBS = 0.26) than the CRS (IBS = 0.30) and TBS (IBS = 0.29), indicating a more accurate prediction of patient outcomes over time (Table 2). Although the C‐index for the Habitat model (0.52) was similar to those of the CRS (0.52) and TBS (0.57), the lower IBS suggests better model calibration and improved long‐term prognostication.

**Table 2:** Comparison of predictive performance among prognostic models for colorectal liver metastases.

Model	C‐index	IBS
CRS	0.52	0.30
TBS	0.57	0.29
Habitat	0.52	0.26

Abbreviations: CRS, clinical risk score; IBS, integrated brier score; TBS, tumor burden score.

### 3.7 Time‐Dependent Predictive Accuracy

**Figure 6:**
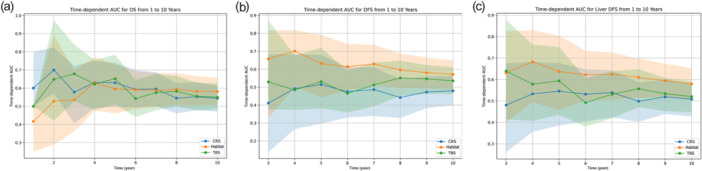
Time‐dependent area under the curve analysis of prognostic models in colorectal liver metastases. (a) Time‐dependent AUC for OS from 1 to 10 years, comparing the predictive accuracy of the CRS, Habitat clusters, and TBS. (b) Time‐dependent AUC for DFS from 1 to 10 years, showing the prognostic performance of CRS, Habitat, and TBS over time. (c) Time‐dependent AUC for liver‐specific DFS from 1 to 10 years, illustrating the predictive power of CRS, Habitat, and TBS for liver‐specific recurrence. The shaded areas represent the 95% confidence intervals for each model. AUC, area under the curve; CRS, clinical risk score; DFS, disease‐free survival; OS, overall survival; TBS, tumor burden score.

The time‐dependent AUC analysis (Figure 6) further demonstrated the superior predictive accuracy of the Habitat model for DFS and liver‐specific DFS over a 10‐year period. The Habitat model consistently outperformed CRS and TBS, particularly in the mid‐range years (3–7 years). This indicates that the Habitat model may be better suited for long‐term prognostication in patients with CRLM, making it a valuable tool for clinicians seeking to personalize treatment strategies based on tumor biology.

### 3.8 NRI and IDI

**Figure 7:**
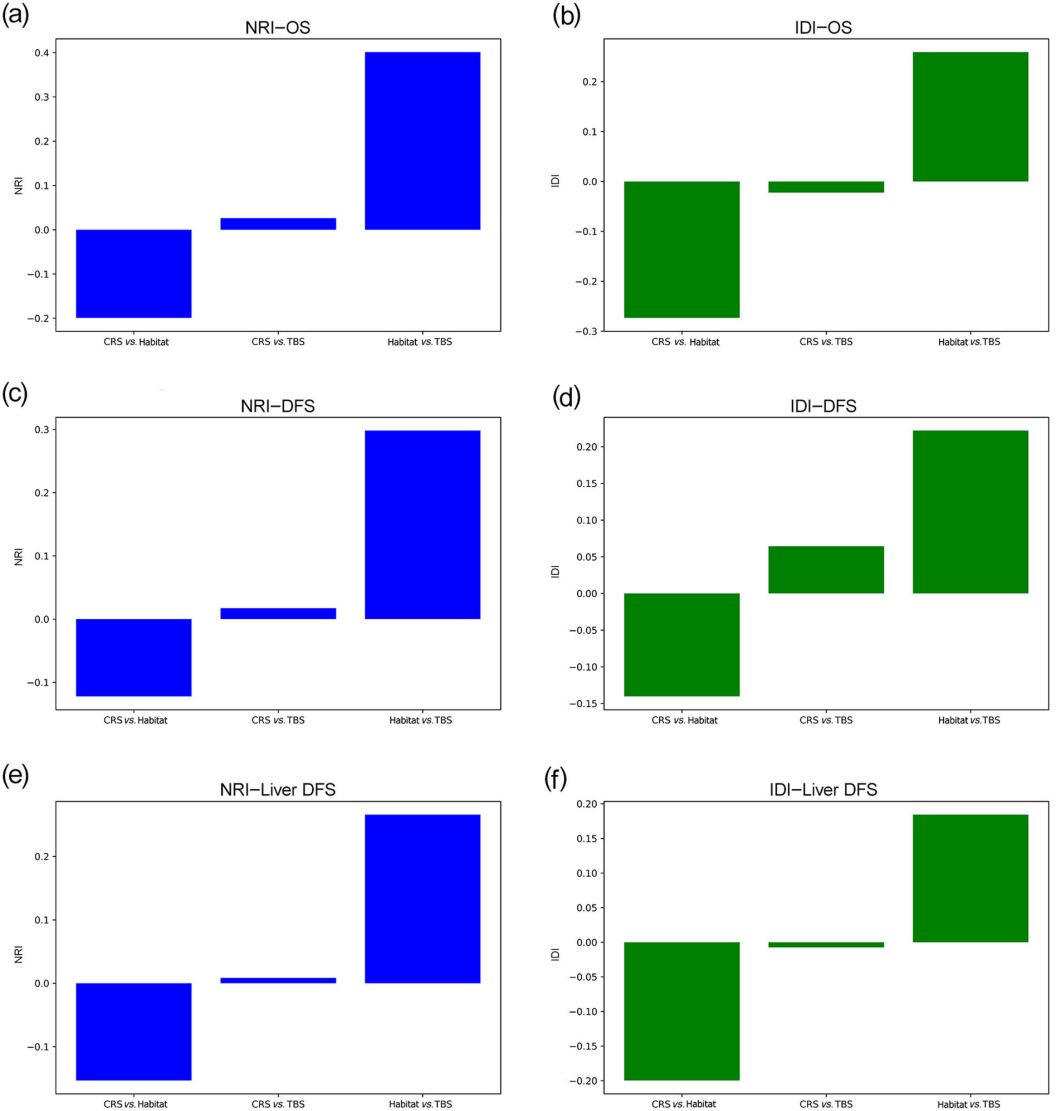
Comparison of NRI and IDI for OS, DFS, and Liver‐Specific DFS. (a) NRI for OS: comparison of CRS versus Habitat, CRS versus TBS, and Habitat versus TBS models. The Habitat versus TBS model showed the highest NRI, indicating significant improvement in reclassification for OS. (b) IDI for OS: Habitat versus TBS also demonstrated the highest IDI, suggesting a marked improvement in discriminative ability for OS prediction. (c) NRI for DFS: Habitat versus TBS outperformed the other models in terms of reclassification improvement for DFS, while CRS versus Habitat showed negative reclassification benefit. (d) IDI for DFS: The Habitat versus TBS model provided the greatest improvement in discriminative ability for DFS, further supporting its superiority in prognosis. (e) NRI for liver‐specific DFS: Habitat versus TBS demonstrated substantial improvement in reclassification for liver‐specific DFS, with CRS versus Habitat again showing negative NRI. (f) IDI for liver‐specific DFS: as for the other outcomes, the Habitat versus TBS model comparison achieved the highest IDI, indicating the Habitat model's strong discriminative performance for predicting liver‐specific DFS. CRS, clinical risk score; DFS, disease‐free survival; IDI, integrated discrimination improvement; NRI, net reclassification improvement; OS, overall survival; TBS, tumor burden score.

The NRI and IDI metrics were used to further compare the prognostic utility of the models (Figure 7). The Habitat model demonstrated significant reclassification and discrimination improvement in comparison with CRS and TBS, particularly for OS, DFS, and liver‐specific DFS. For OS and DFS, the Habitat model had the highest NRI and IDI, indicating its superior ability to correctly reclassify patients into appropriate risk categories. This further supports the clinical relevance of habitat‐based analysis in enhancing outcome prediction in CRLM.

## 4 Discussion

This study demonstrates that CT‐based habitat analysis can significantly enhance prognostic stratification for patients with CRLM by addressing the limitations of traditional models that fail to capture the full extent of tumor heterogeneity. Compared with clinical and pathological‐based models such as the CRS and TBS, the habitat model provides a more comprehensive assessment of spatial tumor heterogeneity, leading to more accurate risk stratification. Specifically, the unsupervised clustering of habitat features successfully divided patients into two distinct groups with significant differences in OS, DFS, and liver‐specific DFS. The habitat model outperformed CRS and TBS in mid‐ to long‐term predictions (3–7 years), with higher time‐dependent AUC values and lower IBS, thereby demonstrating superior predictive accuracy. By incorporating imaging biomarkers, habitat analysis captures the complexity of the tumor microenvironment and independently predicts patient survival beyond traditional clinical factors. The method provides a more personalized tool for treatment decision‐making, addressing the gap left by conventional prognostic tools that overlook tumor microenvironment heterogeneity.

CRLM exhibits intrinsic tumor heterogeneity, which has driven the investigation of various innovative approaches over recent years [21]. In contrast to traditional genomic analysis, radiomics has become a groundbreaking and promising approach for investigating the intricacies of CRLM [22]. In the study by Yoon and colleagues, a machine learning‐based radiomics model applied to liver magnetic resonance imaging data was developed to predict and detect early tumor response to targeted biological therapy in 17 patients with unresectable CRLM. This model demonstrated the potential for improved treatment planning and patient outcomes [23]. Nevertheless, the study did not assess variations in patient outcomes across the recognized groups. In another study, Wang and colleagues applied unsupervised machine learning to preoperative CT radiomics to identify distinct subgroups within patients with colorectal liver metastases with varying prognoses after hepatic resection [24]. Their preliminary exploration successfully identified distinct patient subgroups with varying prognoses, highlighting a novel methodological approach. However, these studies, which are only studies of radiomics features, do not consider heterogeneity within tumors. Han and colleagues employed a distinctive MRI‐based radiomics approach, analyzing multiple tumor habitats to predict histopathological growth patterns in 182 colorectal liver metastases, and found that this provided a more nuanced and accurate prediction model than traditional radiomics methods [25]. Their research introduced a novel approach to traditional radiomics by leveraging multihabitat MRI‐based radiomics analysis, which enabled a more detailed characterization of tumor heterogeneity and led to improved predictive accuracy for histopathological growth patterns in colorectal liver metastases.

In this study, we analyzed the tumor habitat based on CT images, and the extracted habitat features were clustered into two clusters, which showed four differences in their distribution (*p* = 0.041). Significant differences in survival rates can be explained by differences in habitat composition between Cluster 1 and Cluster 2. Patients in Cluster 2 demonstrated significantly longer OS (*p* = 0.002) and better DFS (*p* = 0.008) and liver‐specific DFS (*p* = 0.007). We found that Cluster 2 had a higher proportion of Habitat 3, while Cluster 1 exhibited more aggressive Habitat 5, Habitat 7, and Habitat 8. This spatial heterogeneity reveals the biological complexity within tumors, reflecting the fact that the microenvironment characteristics of different populations may be directly related to the prognosis and may affect the treatment response and metastasis risk of tumors. The high ratios of Habitat 5, Habitat 7, and Habitat 8 in Cluster 1 suggest that these tumor regions may have higher metabolic activity and a more complex microenvironment, such as high levels of angiogenesis and cell proliferation. These areas are often accompanied by hypoxia, which promotes tumor cells to increase their aggressiveness and metastasis through the hypoxia‐inducible factor pathway. Although these features suggest that tumors are more aggressive, they may also respond poorly to certain treatments, resulting in a poorer prognosis. In the comparison of habitat composition between Cluster 2 and Cluster 1, Cluster 2 exhibited a higher proportion of Habitat 3, and this area may represent an area with lower metabolism, less angiogenesis, and less hypoxia. Less heterogeneity means that tumor cells may grow and spread more slowly, indicating areas that may be more sensitive to treatments such as surgery or chemotherapy, leading to better survival. Through multivariate Cox analysis, we further confirmed the independent prognostic value of habitat characteristics. Even after controlling for traditional clinical factors such as tumor size and fibrosis, habitat characteristics were still significant predictors of survival and showed a strong prognostic effect (Figure 4). This finding supports the value of habitat imaging for providing biological information that goes beyond traditional clinical scores, providing a basis for more accurate treatment decisions.

CRS [26] and TBS [27] are recognized prognostic scoring systems for colorectal liver metastases that have been validated in numerous studies, and their efficacy in outcome prediction has been demonstrated. The habitat model created in this study outperformed them in the prediction of OS, DFS, and liver‐specific DFS, implying a more precise prediction capacity for the radiomics technique than that shown by conventional methods. In this study, we comprehensively compared the performance of the habitat model with traditional models (CRS, TBS), and the results showed that the habitat model had higher classification accuracy and better patient risk reclassification ability. In particular, the reclassification and discrimination abilities of the habitat model with respect to OS, DFS, and liver DFS were significantly better than those of CRS and TBS when analyzed using NRI and IDI. These results not only demonstrate the advantages of habitat models in long‐term prognosis but also suggest that they can provide more granular risk stratification for patient populations that are difficult to accurately classify with traditional models. The Sankey plot shows the stratification of patients by the different models and demonstrates that the habitat model was able to identify a subset of patients with better survival prognosis among those at high risk of CRS. Combined with time‐dependent AUC analysis (Figure 6), habitat models are particularly prominent in the medium‐term prediction range of 3–7 years, further indicating that their predictive power for long‐term prognosis is stronger than that of traditional models. In recent years, novel prognostic scoring systems incorporating genetic mutation information [28] have been proposed, and it is claimed that these can improve predictive accuracy for CRLM survival. However, because of the lack of genomic data in our database, a direct comparison between the habitat model studied and these novel prognostic scores was not possible. While previous research has explored potential links between radiomics features and genomic expression levels, it remains unclear whether radiomics adds prognostic value to genomic models.

This study has several limitations. First, its retrospective design limits the ability to establish causal relationships, and the relatively small patient cohort may restrict the generalizability of our findings. Second, while the use of DICOM‐SEG formatted data provided consistency in tumor segmentation, variability in segmentation accuracy across different institutions and imaging platforms remains a potential concern. Additionally, the exclusion of patients with incomplete metadata may have introduced selection bias. The clinical application of habitat imaging requires further validation in prospective studies and across larger and more diverse populations. Finally, while this study focused on CRLM, the broader applicability of habitat imaging to other tumor types or clinical scenarios needs to be explored.

## 5 Conclusions

This study demonstrates that CT‐based habitat analysis offers significant improvements over conventional prognostic models in CRLM. By capturing tumor spatial heterogeneity, the habitat model enhances risk stratification and provides more accurate predictions of survival outcomes. These findings support the integration of habitat imaging into routine clinical practice, offering a path toward more personalized and precise treatment planning for patients with CRLM.

## Author Contributions

**Chaoqun Zhou:** data curation (equal), methodology (equal), writing – original draft (equal). **Hao Xin:** software (equal), visualization (equal), writing – original draft (equal). **Lihua Qian:** data curation (supporting), supervision (equal), validation (equal). **Yong Zhang:** methodology (equal), resources (equal), supervision (equal). **Jing Wang:** software (supporting), supervision (equal), writing – review and editing (equal). **Junpeng Luo:** funding acquisition (lead), supervision (lead), writing – review and editing (lead).

## Ethics Statement

The data used in this study were obtained from the public dataset of TCIA database, which complied with ethical requirements. The original data collection and sharing procedures were conducted in accordance with relevant ethical guidelines and approvals.

## Consent

This study utilized publicly available datasets that are openly accessible. As these are public data, individual informed consent is not required for secondary analysis.

## Conflicts of Interest

The authors declare no conflicts of interest.

## Supporting information

Supporting information.

## Data Availability

Data are openly available in a public repository that issues data sets with DOIs. The data that support the findings of this study are openly available in [TCIA] at https://doi.org/10.7937/QXK2-QG03, reference number [CC BY 4.0].
