# Supplementary figures and images for: Computed Tomography‐Based Habitat Analysis for Prognostic Stratification in Colorectal Liver Metastases
Source: Cancer Innov. 2025 Mar 12;4(2):e70000. doi: 10.1002/cai2.70000 (PMC11897531; doi:10.1002/cai2.70000)

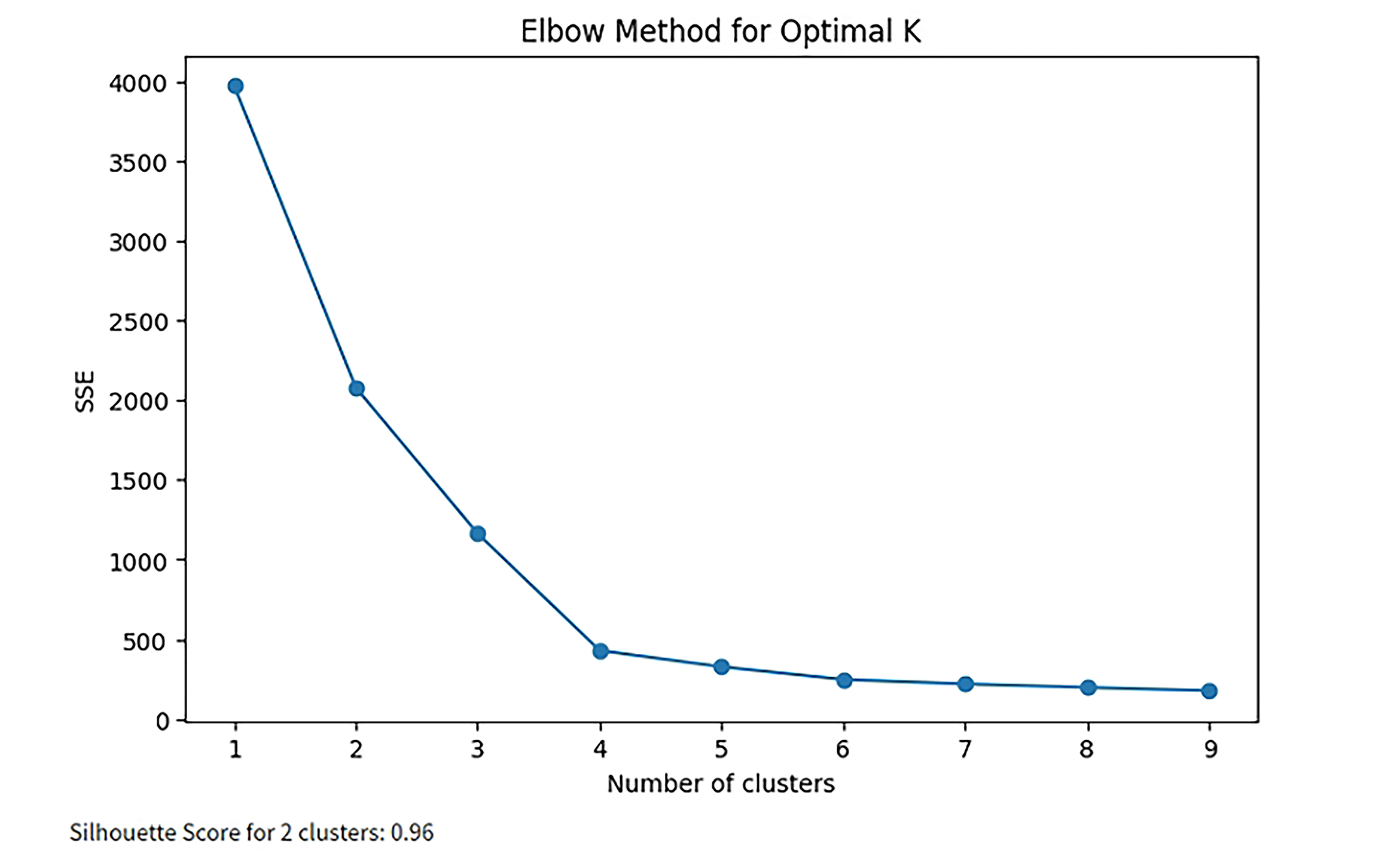

Supplement: Supplementary file 1 — Supporting information. [file CAI2-4-e70000-s001.tif]
